# BOLD Signal Change and Contrast Reversing Frequency: An Event-Related fMRI Study in Human Primary Visual Cortex

**DOI:** 10.1371/journal.pone.0099547

**Published:** 2014-06-12

**Authors:** Pei Sun, Jianfei Guo, Shichun Guo, Jingyi Chen, Le He, Shimin Fu

**Affiliations:** 1 Department of Psychology, School of Social Sciences, Tsinghua University, Beijing, China; 2 Laboratory for Cognitive Brain Mapping, RIKEN Brain Science Institute, 2-1 Hirosawa, Wako, Saitama, Japan; 3 Department of Computer Science, Tsinghua University, Beijing, China; 4 Medical School, Tsinghua University, Beijing, China; 5 Center for Biomedical Imaging Research, Tsinghua University, Beijing, China; University of Montreal, Canada

## Abstract

It is believed that human primary visual cortex (V1) increases activity with increasing temporal frequency of a visual stimulus. Two kinds of visual stimulus were used in the previous studies, one is patterned-flash stimulus with a fixed onset period and an increasing average luminance with the increase of temporal frequency, the other is contrast reversing flickering checkerboard or grating with a constant average luminance across different temporal frequencies. That hemodynamic responses change as a function of reversal frequency of contrast reversing checkerboard is at odds with neurophysiological studies in animals and neuroimaging studies in humans. In the present study, we addressed the relationship between reversal frequency of contrast reversing checkerboard and hemodynamic response in human V1 using an event-related experimental paradigm and found that the transient characteristics of blood oxygenation level dependent response in human V1 depended very little on the reversal frequency of a contrast reversing checkerboard.

## Introduction

A prevailing view from neuroimaging experiments is that human visual cortex increases activity with increasing temporal frequency of a visual stimulus. Using a patterned-flash with a fixed stimulus-on period (5 ms), Fox and Raichle [1] found that the regional cerebral blood flow (rCBF) measured with positron emission tomography (PET) changes in human visual cortex as a linear function of stimulus rate between 0 and 8 Hz, peaked at ∼8 Hz, and decreased at still higher rates. Similar results were also reported by a number of investigators using both PET and functional magnetic resonance imaging (fMRI) [2]–[10]. Subsequently, this stimulus rate (or temporal frequency) dependency was generalized to other types of visual stimuli. In a follow-up study, Fox and Raichle [11] found that changes in rCBF as a function of stimulus rate up to ∼8 Hz were nearly identical for both patterned-flash and contrast reversing checkerboards. As a result, it is often implicitly assumed in functional imaging studies that human visual cortex is optimally activated at a temporal frequency of 8 Hz.

While the stimulus rate dependency with a patterned-flash stimulus can be accounted for by a confound between the average luminance and the stimulus rate, a temporal frequency dependency is surprising for the contrast reversing checkerboard because the average luminance is constant across different reversal frequencies. That hemodynamic responses change as a function of reversal frequency of contrast reversing checkerboard is at odds with neurophysiological studies in cat [12]–[15], ferret [16], [17], and macaque monkey [18], [19] indicating that primary visual cortex (V1) contains two major populations of neurons (low-pass and band-pass neurons). How these two groups of neurons are distributed in the cortex is still under debate [20], with one claiming that they form fine (∼ 0.5 mm in cat V1) columnar structures [21] while the other suggesting that no clear spatial cut between them [19]. In either case, neuroimaging studies would be expected to sample the activity of both populations (at least for low spatial resolution studies) and therefore show similar responses to both high and low temporal frequencies.

In fact, similar activations to low and high temporal contrast reversing frequencies have been observed in many other neuroimaging studies [22]–[37] using either gratings or checkerboard. Because the measurement of brain activation was made mainly by a block-design and was averaged over a certain period of time in these studies, it is still unclear whether there are any differences in the transient aspects of brain activation across different stimulus frequencies. We have addressed this issue by using fMRI with an event-related paradigm and found that there is no clear stimulate rate dependency in the transient characteristics of blood oxygenation level dependent (BOLD) signal on contrast reversing frequencies.

## Materials and Methods

### Subjects

Eight subjects (5 males and 3 females, ages 28–35) with no past history of psychiatric or neurological diseases participated in the experiments, which were approved in advance by the RIKEN Functional MRI Safety and Ethics Committee. The subjects were selected after morphological screening of high-resolution three-dimensional anatomical MR images (1×1×1 mm^3^). To cover targeted cortical area by limited number of slices, the calcarine sulcus of the selected subjects follows a relatively straight course and the cortex around their calcarine V1 is relatively flat and less sulcated in at least one hemisphere, which was then used as the target subsequent study. All subjects had normal or corrected-to-normal vision and gave their written informed consent before each experiment.

### Imaging hardware

All experiments were conducted on a Varian Unity Inova 4 T whole-body MRI system (Varian NMR Instruments, Palo Alto, CA) equipped with a Magnex head gradient system (Magnex Scientific Ltd., Abingdon, UK). High-resolution three-dimensional T1-weighted anatomical MR images were acquired with a bird-cage radio-frequency (RF) coil. A 5-inch transmit/receive butterfly quadrature RF surface coil was used to acquire functional and co-registered anatomical images in the functional experiments. The surface coil was mounted on a bakelite support frame attached to the patient table.

During the experiment, the subject was made to lie supine on the patient table and to rest the back of the head on the surface coil. Head motion was restricted using a bite-bar as well as padding spongy rubber around the subject's head. The subject's heartbeat was monitored with a pulse oximeter, and respiration was monitored with a pressure sensor placed on the abdominal region. Both signals were recorded along with the timing of RF pulses for later correction of physiological fluctuations.

### Visual stimulation

The presentation of visual stimuli was controlled by a custom-built software package run on a PowerMac G4 computer. Stimuli were delivered to the subject's eyes via an optic fiber goggle system (Avotec, Inc., Jensen Beach, FL) that subtended 25°×19° of visual angle. The image on the goggles had a resolution of 800×600 pixels and a refresh rate of 60 Hz. The subject adjusted two refractive correction lenses on the goggles to achieve corrected-to-normal vision. The luminosity of the brightest white available on the goggles was ∼4 lux and the darkest black was ∼0.5 lux. Image acquisition and stimulus presentation times were synched and the trigger signal, when needed, was sent from the scanner's console to the PowerMac G4 computer equipped with a digital I/O board (PCI-DIO-96, National Instruments, Austin, TX). All signals related to the timing of RF pulses, stimulus onset and offset, as well as the state of the subject, including physiological data, behavioral performance, head position and eye position, were simultaneously recorded using a PowerMac G3 computer with a data acquisition board (PCI-6023E, National Instruments, Austin, TX) and a custom-built program written in LabView (National Instruments, Austin, TX).

In the experiments, a circular black/white contrast reversing checkerboard, which consisted of checkers of ∼0.8° and had a diameter of 7.6°, was placed 7.6° diagonally away from the fixation cross. We presented the checkerboard in the lower-left or lower-right quadrant, depending on the targeted hemisphere to be studied, and avoided the fovea and the horizontal and vertical meridians to confine the activation within circumscribed retinotopic areas (only V1 was studied in this study). The checkerboard was temporally modulated in a square wave, and the contrast of the checkerboard within a scan was reversed at a given frequency (0.17, 0.75, 2, 4, 8, or 15 Hz). The contrast reversal frequency was defined as square-wave modulation cycles of contrast. At a frequency of 1 Hz, therefore, each square in the checkerboard would be displayed in both black and white during one second.

Special considerations were taken in choosing the frequencies tested. First, since our custom-built program presented the stimulus exactly at the beginning of each frame (with a refresh rate of 60 Hz), the highest achievable reversal frequency with precise timing using our stimulation presentation device was 15 Hz (containing 30 contrast transitions). Second, because signal fluctuations caused by cardiac pulsations (∼1 Hz) were removed in the data post-processing, a low frequency of 0.75 Hz, instead of 1 Hz, was used. Third, for comparison with previous studies, a very low frequency was introduced. Because the brief stimulus presentation time (3 s) as required by the event-related design, the lowest contrast reversal frequency can be achieved is 0.17 Hz, corresponding to the appearance and disappearance of the checkerboard. Finally, it should be pointed out that the liquid crystal display (LCD) projector or goggle, compared with conventional cathode ray tube (CRT) monitors, has relative slow rise- and fall-times [38]. As a result, the luminance contrast of our display (LCD goggle) decreases as temporal frequency increases. The rise and fall-times of our display were measured and this confounding tendency was corrected accordingly [28], and a fixed contrast level (33%) was used for all the frequencies.

We also made efforts to restrict our analyses to the gray matter of a well-defined, circumscribed portion of V1. In all six scans, the checkerboard was confined to a quadrant of the visual field. This stimulus configuration, combined with the meridian mapping that demarcated V1/V2 borders of individual subjects (see below), allowed us to localize activation in the expected portion of V1.

Throughout the experiment, the subject was instructed to fixate on the central fixation cross (in unsaturated red). To help maintain fixation and the level of arousal, the subject performed a detection task on the fixation cross. Every 2–5 s, the luminance of the fixation cross was increased for 500 ms, and the subject was to report that event with a button press. Data would be discarded once the subject missed to press the button more than 5%. We nevertheless monitored the subject's eye position using iView (SensoMotoric Instruments, Boston, MA) and the eye tracker built into the goggle system. Offline analysis confirmed that the subject performed the task correctly more than 99% and maintained fixation throughout the experiment and therefore no data were discarded.

### Imaging parameters and experimental paradigms

In addition to screening the morphology around the calcarine sulcus, we also determined the V1/V2 borders of individual subjects using a standard method for mapping the representation of the vertical meridian [39], [40]. The V1/V2 borders were determined in a separate meridian and retinotopic mapping experiment. In the meridian and retinotopic experiment, there were two blocked-design runs in which a wedge (15°) of checkerboard was presented 6 times at vertical or horizontal meridian with 30 s on and 30 s off periods and six traveling wave design runs in which a wedge (90°) of checkerboard with rotating speed in 24 s/cycle was presented 12 times. Based on the mapped V1/V2 borders, in the main functional experiment, the slices were placed parallel to the calcarine sulcus on the target hemisphere to completely contain the dorsal V1, with one optimally covering the dorsal bank of the calcarine sulcus. Prior to each functional experiment, T1-weighted anatomical images at the same slice positions as functional images were acquired with a four-segment inversion recovery FLASH pulse sequence (slice thickness  =  3 mm; FOV = 24×24 cm^2^; in-plane resolution  =  0.94×0.94 mm^2^; volume TR = 1. s; TE = 10 ms; number of averaging  =  2).

In the functional experiment, five contiguous slices (slice thickness  =  3 mm; FOV = 24×24 cm^2^; in-plane resolution  =  3.75×3.75 mm^2^) were collected. Functional images were acquired with a two-segment centric-ordered EPI pulse sequence (volume TR = 0.5 s; TE = 25 ms; average FA = 45°). Twelve trials, each consisting of a 3 s stimulation period with a checkerboard reversing at one of the six frequencies and a 30 s baseline period with a homogeneous gray screen, were repeated. Before the first trial, a 33 s baseline was inserted. In total, 858 volumes over 429 seconds were acquired in a scan. Six scans, each with a reversal frequency chosen in pseudo-randomized order, were collected from each subject. In addition, before the six event-related scans, a region of interest (ROI) scan was collected with identical imaging parameters and slice prescription on each subject. During the scan, in which 360 volumes over 180 seconds were collected, the subject viewed three cycles of a 2 Hz checkerboard and a gray screen. The ROI identified in this scan was used for quantifying BOLD responses in subsequent event-related scans.

### Data processing and analysis

In all functional experiments, longitudinal magnetization was allowed to reach steady state before EPI images were collected. EPI distortions were minimized using a reference volume (without phase encoding) acquired at the beginning of each experiment [41]. The first echo in each segment was a navigator echo, which was used to correct inter-segment phase and amplitude variations [42]. After EPI images were reconstructed, cardiac and respiratory fluctuations were removed from time series images using a retrospective estimation and correction method which employed pulsation and respiration data recorded during image acquisition [43]. Two-dimensional motion correction was also applied [44]. Both corrections were applied in k-space. A high-pass filter was used to suppress baseline signal drifts. This filter was set to have a 3dB drop-off cutoff frequency of 0.00085 cycles/sec but to retain the DC level. No other spatial or temporal smoothing was applied. Statistical analyses were performed using the software package STIMULATE developed at the University of Minnesota (J.P.Strupp, NeuroImage 3, S607, 1996). Activated voxels (displaying positive signal changes) were identified by the Student's t test comparing images acquired during stimulation periods and those acquired during control periods on a voxel-by-voxel basis. To account for the hemodynamic delay, the first image acquired in each period in the block-design experiments was excluded.

Due to large geometrical variations around V1 between the subjects, quantitative analyses for data from the experiments were performed on a single-subject basis. In the event-related experiment, a time course (3 s checkerboard stimulation and 30 s gray screen for each trial, 12 repetitions) for each of the six frequencies was obtained by averaging BOLD responses from all the voxels within the ROI defined in the preceding ROI scan. The baseline (0% BOLD response) was determined from the 33 s control period before the first trial. To quantify the transient BOLD response to each frequency, we first fit each time course using a gamma function:



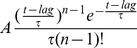
(1)where A is the amplitude; n is a shape parameter that was allowed to take on integer values from 4 to 6; and τ roughly corresponds to the width of the response and was allowed to take on values from 0.5 to 2 seconds. The lag value controls when the gamma function begins relative to stimulus onset and take on values ranging from 0 to 4 seconds. When t-lag was negative, the function was set to 0. A difference of gamma functions, in which the second gamma function was allowed to have τ values between 0.5 and 4 s and a lag 2–8 s, was used to capture the delayed negative response of the hemodynamic function. Levenberg-Marquardt optimization was used to find the parameters of the two gamma functions that minimized the mean squared difference between the estimated hemodynamic response and the actual time course. From the estimated hemodynamic response for each of the six frequencies, we extracted four parameters, that is, the maximum BOLD response, full-width at half-maximum (FWHM) of BOLD response curve, integrated area under the BOLD response curve (for this calculation, we only used positive responses; post-stimulus undershoots were not used) and time to reach maximum BOLD response, and compared them across the eight subjects. All data analyses mentioned above and further statistical analyses were performed using in-house developed and built-in functions in Matlab (The MathWorks, Inc., Natick, MA).

### Control experiment

In addition to the main experiment described above, a control experiment was run with a different group of subject, different settings of imaging hardware, and a different experimental paradigm. Specifically, four subjects (3 males and 1 female, ages 19–28) with no past history of psychiatric or neurological diseases participated in the experiments, which were approved by the Tsinghua Functional MRI Safety and Ethics Committee. The experiment was conducted on a Phillips Achieva 3 T MRI system at the Center for Biomedical Imaging Research, Tsinghua University. High-resolution three-dimensional T1-weighted anatomical MR images, functional and co-registered anatomical images were acquired with a 32-channel RF coil. Visual stimulation was delivered to the subject's eyes via a mirror and a screen system (Invivo, Gainesville, FL). Same contrast level across different temporal frequency was achieved using a psychophysical procedure [45] based on the prior measurements of rising and falling characteristic of LCD monitors [28]. In the control experiment, there were three long runs. In each long run, twenty-four trials were conducted and one trial consisted of a 3 s stimulation period and a 30 s baseline. Six reversing frequencies were repeated 4 times in random order in the long run. Before the first trial, a 33 s baseline was inserted. To avoid selection bias, 6 Hz instead of 2 Hz was used in the localizer experiment, which was a different frequency from that used in the main experiment and in the middle range of all the six temporal frequencies. In the control experiment, seven contiguous slices (slice thickness  =  3 mm; FOV = 22×22 cm^2^; in-plane resolution  =  2.75×2.75 mm^2^) were collected. Functional images were acquired with a single-shot EPI pulse sequence (TR = 0.5 s; TE = 35 ms; FA = 90°). Functional data were pre-processed (i.e., head motion correction and baseline drifts suppression) using AFNI (http://afni.nimh.nih.gov/afni/).

## Results

In the fMRI experiment, we investigated several transient aspects of BOLD responses to the reversal frequency by quantifying four parameters, namely, the maximum BOLD response, full-width at half-maximum (FWHM) of BOLD response curve, integrated area under the BOLD response curve and time to reach maximum BOLD response in a circumscribed region of V1 following a brief exposure (3 s) to a black/white checkerboard whose contrast was reversed at 0.17, 0.75, 2, 4, 8, or 15 Hz. Each frequency in the experiment was studied in a separate scan.

Before the main event-related experiment, a functional localizer experiment was conducted with a block-design for each subject using the frequency of 2 Hz that is in the middle of used frequencies. Based on the statistical t-map (p<0.01) obtained in the functional localizer experiment, a region of interest (ROI), which extended across 2 to 3 slices, depending on the morphology of V1 of individual subjects, was determined. The same ROI was then used for quantitative analysis for all the frequencies in the event-related experiments. Overall, about 8 to 15 activated voxels were found for each subject by this procedure.

**Figure 1:**
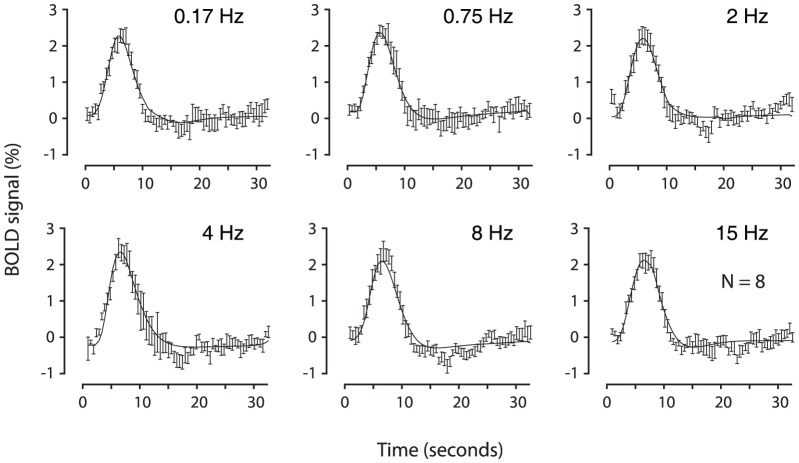
Hemodynamic responses to six reversal frequencies examined in an event-related experiment. The symbols and error bars in each panel (mean ± SEM) show the average of original time course obtained from eight subjects. The solid curve in each panel shows the estimated hemodynamic response averaged from eight subjects (for clarity, error bars of estimated hemodynamic response are not shown). To quantify hemodynamic responses to different reversal frequencies, four parameters were extracted and are given in Table 1.

To quantify the dependency of the transient BOLD response on reversal frequency, we obtained the estimated hemodynamic response by averaging the time course of all the voxels inside ROI for each of the six frequencies (**Figure** **1**), from which we extracted four parameters, the maximum BOLD response, full-width at half-maximum (FWHM) of BOLD response curve, integrated area under the BOLD response curve and time to reach maximum BOLD response (**Table 1**). A one-way repeated-measures ANOVA revealed no frequency effect (p = 0.699, maximum BOLD response; p = 0.213, FWHM of BOLD response curve; p = 0.877, integrated area under the BOLD response curve; and p = 0.429, time to reach maximum BOLD response). Please note that almost same amplitudes of BOLD response were already observed at low frequencies as that at high frequencies (e.g., 0.17 Hz elicited ∼93% of response by 8 Hz).

**Table 1:** Parameters extracted from estimated hemodynamic responses to six temporal frequencies in an event-related experiment.

Frequency (Hz)	Max value (%)	FWHM (s)	Area (% × s)	TTM (s)
0.17	2.26±0.19	5.00±0.30	25.54±3.22	5.66±0.16
0.75	2.39±0.21	5.44±0.52	30.08±3.79	5.38±0.23
2	2.31±0.32	5.42±0.27	29.24±4.54	5.75±0.23
4	2.57±0.22	4.83±0.28	27.82±3.38	5.71±0.33
8	2.45±0.23	5.15±0.27	26.72±3.29	5.99±0.27
15	2.33±0.23	5.76±0.24	27.73±2.11	5.59±0.28

Data were obtained from eight subjects (five right and three left V1). All values are given in mean ± SEM. Frequency: contrast reversal frequency (Hz); Max value: maximum BOLD response in percent (%); FWHM: full-width at half-maximum of BOLD response curve in second (s); Area: integrated area under the BOLD response curve in percent multiplied by second (% × s); TTM (s): time to reach maximum BOLD response in second (s).

It is known that attention or anticipation may influence the BOLD response in V1 [46] [47]. To rule out this possibility, we did a control experiment with a different paradigm (see **Materials and Methods**). In the control experiment, there were three long scans. In each long scan, all six temporal frequencies were tested 4 times in random order. In addition, to avoid selection bias, 6 Hz instead of 2 Hz was used in the localizer experiment. Similar result was obtained from four new subjects (**Table 2**), a one-way repeated-measures ANOVA showed that there were no significant differences in the transient characteristics of BOLD responses under different reversal frequencies (p = 0.271, maximum BOLD response; p = 0.823, FWHM of BOLD response curve; p = 0.217, integrated area under the BOLD response curve; and p = 0.780, time to reach maximum BOLD response).

**Table 2:** Parameters extracted from estimated hemodynamic responses to six temporal frequencies in the control experiment from another four subjects (three right and one left V1).

Frequency (Hz)	Max value (%)	FWHM (s)	Area (% × s)	TTM (s)
0.17	2.87±0.19	5.60±0.42	34.21±3.81	5.56±0.40
0.75	3.08±0.43	6.16±0.26	40.99±6.41	4.99±0.52
2	4.08±0.54	5.99±0.16	56.93±11.23	5.30±0.42
4	3.64±0.52	6.02±0.42	45.46±6.22	5.54±0.53
8	4.23±0.62	6.19±0.38	55.15±8.80	5.72±0.32
15	3.31±0.35	5.72±0.45	40.31±2.30	5.85±0.43

See conventions in **Table 1**.

## Discussion

Using a spatially confined black/white contrast reversing checkerboard that activated a circumscribed portion of early visual cortex, we found that the transient characteristics of BOLD response in human V1 depended very little on the reversal frequency. The results from the present and our former study [28] appear to be at odds with the prevailing thought that hemodynamic responses depend on the temporal frequency of visual stimuli. However, a careful review of the literature reveals that the discrepancy stems largely from the types of stimuli used in various experiments.

In their seminal PET study, Fox and Raichle [1] first demonstrated stimulus rate dependency of rCBF changes in human visual cortex (around V1) using patterned-flash stimuli. Specifically, they showed that rCBF change was a linear function of stimulus repetition rate between 0 and 8 Hz, peaked at ∼8 Hz, and then declined as the rate further increased. Similar observations were also made subsequently in a number of studies using both PET and fMRI [2]–[10]. The stimuli used in all these studies, including the original one by Fox and Raichle, were flashing strobe lamps, checkerboards or light-emitting diodes (LEDs) with a flash duration ranging from 50 µs to 5 ms. The stimulation rate (or temporal frequency), therefore, was the number of flashes per second, and the luminance averaged over the entire stimulation period was a function of stimulus rate. In this sense, the observed rCBF change or the BOLD response following stimulation with patterned-flash stimuli most likely reflects the average luminance during stimulation rather than the temporal frequency, though, above 8 or 15 Hz, it is not clear why the responses saturate.

A contrast reversing checkerboard (contrast reversals between black and white checkers at equal intervals), however, is very different from patterned-flash stimuli (flashing checkers or LEDs) because the average luminance during the stimulation period is constant across different reversal frequencies. That rCBF changes as a function of stimulus rate were nearly identical for both patterned-flash stimuli and contrast reversing checkerboard was claimed in a follow-up study by Fox and Raichle [11]. However, the data point for the contrast reversing checkerboard at 0 Hz in that study was from a scan without visual stimulation, instead of a scan using a stationary checkerboard that would have maintained the same stimulus luminance as with other frequencies. Naturally, this 0 Hz condition defined for the contrast reversing checkerboard, which physically was identical to the 0 Hz for the patterned-flash, also produced an rCBF change near the baseline. Thus, the apparent similarity of rCBF changes between 0 and 7.8 Hz for the two types of stimuli was most likely due to this inappropriate use of stimulus for the 0 Hz contrast reversing checkerboard. Indeed, inspecting their figure reveals that the rCBF change using the contrast reversing checkerboard at 1 Hz (the lowest frequency actually tested) was ∼65% of the maximal response observed at 7.8 Hz, whereas the change using the patterned-flash at 1 Hz was only ∼17% of the maximum.

Despite the fact that a linear relationship between rCBF or BOLD response and the reversal frequency of contrast reversing checkerboard has never been established, such a frequency dependency is often implicitly assumed in various neuroimaging studies when contrast reversing checkerboards are used. The results from several other studies, however, have provided very little support for such a relationship. In a parametric study using ^1^°CO_2_ PET, Law and colleagues [24] measured rCBF changes following a contrast reversing checkerboard stimulation with 10 reversal frequencies from 0.03 to 30 Hz, and found that the maximal response, observed between 6 and 15 Hz across subjects, displayed only a small increase (8%) from that observed at 0.03 Hz. Similarly, in fMRI experiments, Kastner and colleagues [22] reported that the BOLD response to 0.5 Hz was ∼84% of the maximal response elicited by 20 Hz; Muthukumaraswamy and colleagues [33] showed that BOLD response to 0 Hz was ∼65% of the maximal response elicited by 16 Hz under two different spatial frequencies (3 cpd and 0.5 cpd). Moradi and colleagues also made the observation that BOLD responses to reversal frequencies of 0.71 and 8 Hz were qualitatively similar [25, see their Figures 8B and 8C]. As different protocols were used in previous studies and there are essential difference between rCBF and BOLD signal, how rCBF or BOLD response in human V1 changes with the reversal frequency of contrast reversing checkerboard need to be further investigated.

Finally, it should be pointed out that the observed hemodynamic activity in the current study is a combination of the impulse response to the sudden presentation of visual stimulation that included many visual features (e.g., spatial frequency, orientation, contrast, etc.) and the neural response to certain temporal frequency during the brief stimulation period (i.e., 3 seconds). Given that the difference of BOLD responses between different pattern-flash frequencies could be detected using a briefer stimulation (i.e., 1 second) in a former study [5], we may claim that such difference is not existed or at least the tendency is not evident enough with the current experimental settings. Furthermore, even we could extend the duration of visual stimulation, another confound factor, that is the visual adaptation, which typically results in reduction in neuronal response over prolonged exposure to a visual stimulus, will be introduced and this extension will lead to a block-design experiment eventually. Taken the results using the block-design in our former study [28] and the results using slow event-related design in the present study, we conclude that there is no clear stimulate rate dependency in the transient characteristics of BOLD signal on contrast reversing frequencies.
